# Perioperative biologic use and postoperative outcomes in patients with inflammatory arthritis: a systematic review

**DOI:** 10.1186/s41927-026-00650-y

**Published:** 2026-04-30

**Authors:** Samantha Brady, Johanna Taylor, Katie Carlisle, Joy Adamson, Helen Fulbright, Catherine Hewitt, Laura Mandefield, Kulveer Mankia, Hemant Pandit, Andrew Mott

**Affiliations:** 1 York Trials Unit, Department of Health Sciences, University of York, York, Heslington YO10 5DD UK; 2 Centre for Reviews and Dissemination, University of York, York, UK; 3 Leeds Institute of Rheumatic and Musculoskeletal Medicine, University of Leeds, Leeds, UK; 4 NIHR Leeds Musculoskeletal Biomedical Research Unit, Leeds, UK

**Keywords:** Inflammatory arthritis, Biologics, Surgery, Perioperative, Systematic review, Wound healing, Surgical site infection, Disease flare, Quality of life

## Abstract

**Objectives:**

To evaluate the impact of continuing versus stopping biologic disease-modifying anti-rheumatic drugs (bDMARDs) during the perioperative period on surgical site infections (SSIs), delayed wound healing, and disease flares in patients with inflammatory arthritis (IA) undergoing elective non-orthopaedic surgery.

**Methods:**

We conducted a systematic review of observational studies assessing the perioperative management of bDMARDs in IA patients undergoing elective non-orthopaedic surgery. Searches were conducted across seven databases and trial registries from the year 2000 onwards. Eligible studies compared outcomes in patients who continued versus stopped biologic therapy. Risk of bias was assessed using the ROBINS-I tool and due to heterogeneity data were synthesised narratively.

**Results:**

Eight observational studies met the inclusion criteria. All studies were at moderate or serious risk of bias. Four studies compared infections with two suggesting higher infection rates in the stop biologic group. Two studies assessed delayed wound healing, both suggesting higher rates in the stop group. Disease flares were more common in patients who stopped biologics in all three of the studies reporting this outcome. No studies assessed health-related quality of life.

**Conclusions:**

The available observational evidence does not demonstrate a consistent increase in postoperative infection or wound complications related to biologic continuation, however, confidence in these findings is limited by serious risk of bias and outcome heterogeneity. High-quality randomised controlled trials are needed to inform the perioperative management guidelines for IA patients undergoing elective non-orthopaedic procedures.

**Supplementary Information:**

The online version contains supplementary material available at 10.1186/s41927-026-00650-y.

## Introduction

Inflammatory arthritis (IA) is a group of different conditions including rheumatoid arthritis, psoriatic arthritis, axial spondyloarthritis, and juvenile inflammatory arthritis. Biologic Disease Modifying Anti-Rheumatic Drugs (bDMARDs or biologics) are commonly used groups of medications which reduce inflammation in these conditions by targeting the body’s immune response. These medications work through acting on different targets, with the aim of reducing disease flares and managing symptoms [1].

When patients have surgery they are at risk of infection, and patients on biologics already have a higher risk of infection compared to those not receiving biologics [2]. It has therefore become standard practice internationally to stop biologics ahead of surgery and the existing guidelines from the British Society of Rheumatology [3] and the American College of Rheumatology [4] recommend stopping biologics. However, patients who stop their biologics are at risk of a disease flare of their IA [5].

A systematic review comparing the evidence for stopping versus continuation of biologics in the perioperative period in orthopaedic surgery found that there was limited evidence for increased risk of developing infection or wound complications with continuation [6]. However, they also noted that the retrospective and heterogeneous nature of the data made it difficult to draw conclusions on the best clinical practice. The ongoing PERISCOPE randomised controlled trial [7] is investigating whether continuation versus stoppage of biologics in patients with IA undergoing elective orthopaedic surgery is clinically effective with regards to health-related quality of life.

There is no existing systematic review summarising the evidence for continuation versus stoppage of biologics for patients with IA in non-orthopaedic surgery. The existing clinical guidelines are primarily based on orthopaedic surgery and acknowledge that there is limited evidence regarding the associated risks of infection, wound healing, and flares [3, 4].

## Aims & objectives

To undertake a systematic review comparing the stopping or continuation of biologics in the perioperative period for IA patients undergoing non-orthopaedic surgery. The following outcomes of interest will be assessed: infections, delayed wound healing, disease flares, and quality of life.

## Methods

### Registration

The review was registered on PROSPERO on 14th April 2023 (CRD42023416888).

### Eligibility criteria

Studies were eligible for inclusion in this review if they met the following criteria:

#### Participants

Patients with IA on bDMARDS undergoing elective non-orthopaedic surgery.

#### Intervention

Continuation of bDMARDS throughout the perioperative period.

#### Comparator

Stopping bDMARDS in the perioperative period (current practice).

#### Study design

Randomised controlled trials, non-randomised studies, or any experimental or observational study design were eligible to be included in this review.

An initial search with the study design limited to randomised controlled trials did not identify any relevant studies during the screening phase. Consequently, the eligibility criteria were broadened to include all types of observational study designs.

### Electronic searches

An Information Specialist (HF) designed a preliminary search for Ovid MEDLINE, in consultation with the review team. This search strategy was then translated for use in bibliographic databases using relevant subject headings (controlled vocabularies) and search syntax, appropriate to each resource.

The search strategies were designed to identify non-randomised studies on the use of biologic drugs during the perioperative period for patients with IA. Searches were limited to being published from 2000 onward. No restrictions on language were applied to the searches. See Additional File 1 for full search strategies.

The results of the database searches were deduplicated in EndNote 21.

The following databases and trial registries were searched on 23rd April 2024:

1. MEDLINE(R) ALL (Ovid) (1946 to April 22, 2024);
2. Embase (Ovid) (1974 to 2024 April 22);
3. Cochrane Central Register of Controlled Trials (Wiley): 2024, Issue 3 in the Cochrane Library;
4. CINAHL Complete (EBSCO) (Inception - Current);
5. International HTA database (https://database.inahta.org/search/advanced) (Inception - Current);
6. ClinicalTrials.gov (US NIH), (all available years);
7. International Clinical Trials Registry Platform (WHO), (all available years).

Using a seed set of 12 records [8–19], the following sources and citation indexes were searched both forwards and backwards on 4th November 2024:

- Web of Science Core Collection (Science Citation Index Expanded (SCI-Expanded) 1900-present; Social Sciences Citation Index (SSCI) 1956-present; Arts & Humanities Citation Index (AHCI) 1975-present; Conference Proceedings Citation Index – Science (CPCI-S) 1990-present; Conference Proceedings Citation Index – Social Science & Humanities (CPCI-SSH) 1990-present; and Emerging Sources Citation Index (ESCI) 2015-present) via Clarivate https://webofscience.com
- Citation Chaser via Shiny Apps https://estech.shinyapps.io/citationchaser/

Citations were added to EndNote 21 (Clarivate Analytics) and deduplicated.

### Screening and data extraction

Titles and abstracts were initially screened by two reviewers, followed by the retrieval of full texts for the remaining records using Covidence screening software [20]. Disagreements were resolved through discussion amongst the wider team.

For studies meeting the inclusion criteria, data were extracted by two researchers independently using a study specific data collection form. The data items included: author, study objective, design, funding source, participant and intervention characteristics (including eligibility criteria), methods of infection assessment, indicators of disease activity, quality of life measures, and outcomes related to delayed wound healing.

Two reviewers independently assessed the risk of bias of the included studies using the Risk Of Bias In Non-randomised Studies - of Interventions (ROBINS-I v2) tool [21]. Disagreements were resolved by discussion. The risk of bias assessments were visualised using the robvis web tool [22].

### Data synthesis

Key study and participant characteristics, and study quality are summarised narratively and within tables. We planned for studies to be pooled in a fixed or random effects meta-analysis depending on the outcome and extent of heterogeneity. However, insufficient data were available to meta-analyse due to the small number of included studies and heterogeneity in terms of the included patient populations. We therefore provide a narrative summary of each of the outcomes.

## Results

**Fig. 1:**
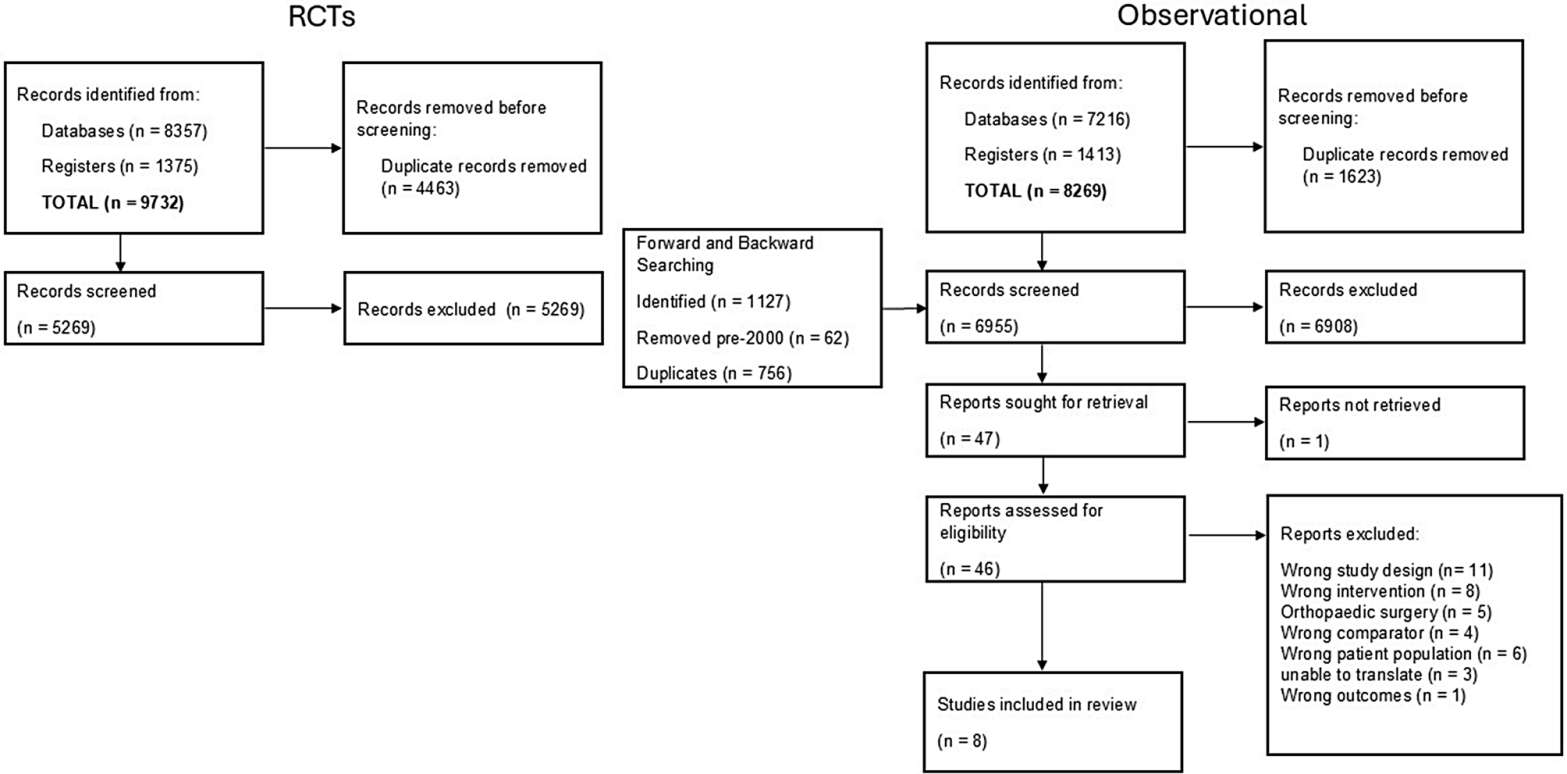
Biologics review paper flowchart_updated following reviewers comments

A total of 6646 records were identified following de-duplication through database searches. Of the 1127 records retrieved during citation searching, 62 records were removed with a publication date prior to 2000. A total of 309 records remained after de-duplication. Following the initial screen, 46 full texts were assessed with eight being eligible for inclusion [8–12, 16, 19, 23]. Full details of the screening process are detailed in the PRISMA diagram (Fig. 1). The full list of excluded studies with reasons is included in Additional File 2.

All included studies were retrospective in design, consisting mainly of retrospective cohort studies, along with a small number of retrospective observational, cross‑sectional and service audit studies.

Most of the included studies involved mixed surgical populations including orthopaedic procedures without stratified outcome reporting. Whilst the initial aim of the systematic review was to compare the stopping or continuation of biologics in the perioperative period for IA patients undergoing non-orthopaedic surgery, it was decided that restricting inclusion only to studies reporting stratified non-orthopaedic outcomes would result in the exclusion of the majority of available evidence and substantially reduce the comprehensiveness of the review.

Reporting on stoppage of biologics varied widely across studies. Where details were provided, stoppage of biologics ranged from a median of 3 weeks to last infusions occurring 4–8 weeks, or up to 6 months, before surgery. One study categorised patient’s as having ‘interrupted therapy’ if biologics were stopped for more than five half‑lives, while another required stoppage within defined dosing intervals. Overall, only a minority of studies provided clear or consistent information on how long biologics were stopped prior to surgery. Furthermore, the surgical procedures reported across studies were highly variable, with several involving urgent or unplanned operations. This distinction is important, as these cases may not have reflected the perioperative timelines typical of elective surgery.

A summary of the study characteristics is presented in Table 1.

**Table 1:** Study characteristics

Study Details	Patient population & surgical procedures	Steroids	Comorbidities	Age	Smoking	bDMARDS	Perioperative antibiotics	Numbers in stop or continue groups
AbouZahr 2015 [11]	Diagnosis of Rheumatoid Arthritis Any surgery	*n* = 304 participants taking bDMARDs on a chronic steroid (*n* = 187 continued BA, *n* = 93 stopped BA before surgery, *n* = 24 stopped BA after surgery)	Charlson Comorbidity Score - 0-483 (53.2%) 1-208 (22.9%) 2-117 (12.9%) 3–47 (5.2%) 4–27 (3.0%) 5–12 (1.3%) 6 − 5 (0.6%) 7 − 6 (0.7%) 8 − 3 (0.3%)	Below 40: *n* = 21 41–60: *n* = 405 Above 60: *n* = 469	*N* = 252 (28.1%)	Etanercept, Adalimumab, Infliximab, Certolizumab, Golimumab, Abatacept, Anakinra	NR	Stop: *n* = 279 Continue: *n* = 517
Bakkour 2016 [8]	Diagnosis of Psoriasis or Psoriatic Arthritis Skin surgery *n* = 40 (52%), Orthopaedic surgery *n* = 15 (19.5%) Cardiovascular surgery *n* = 6 (7.8%), Gastrointestinal and urologic surgery *n* = 12 (15.6%) ENT, Maxillofacial and dental surgery *n* = 4 (5.2%)	NR	Patients were on concomitant immunosuppressive therapies or had a diagnosis of diabetes *n* = 25 (32%) and *n* = 11 (14%)	While the full cohort mean is not stated, the ages of patients experiencing complications ranged from 34 to 77 years	Patients were smokers in 17 procedures (22%) respectively.	Tumour Necrosis Factor (TNF) inhibitors (Adalimumab, Etanercept and Infliximab; in addition to Golimumab and Certolizumab for PsA alone) and the Interleukin (IL) 12/23 Inhibitor Ustekinumab.	Prophylactic antibiotics were prescribed in four procedures (5%)	Stop: *n* = 20 Continue: *n* = 57
Elia 2020 [16]	Diagnosis of Rheumatoid Arthritis Underwent posterior arthrodesis of the craniovertebral junction	NR	26.3% of patients in the group that continued DMARD therapy had a diagnosis of diabetes, whereas no patients in the discontinued group were diabetic	Total M = 65.2 (SD = 11.19) Continue: M = 65.9 (SD = 12.60) Stop: M = 64.5 (SD = 9.95)	Total *n* = 8 (20.5%) were smokers Stopped BA *n* = 5 (25%) Continued BA *n* = 3 (15.8%)	Methotrexate Leflunomide Abatacept Etanercept Sulfasalazine Hydroxychloroquine Adalimumab Mesalamine Azathioprine Infliximab	NR	Stop: *n* = 20 (5 on biologics) Continue: *n* = 19 (5 on biologics
George 2020 [12]	Diagnosis of Rheumatoid Arthritis Hip fracture repair, abdominopelvic surgery (cholecystectomy, hysterectomy, hernia surgery, appendectomy, colectomy for diverticular disease) or cardiac surgery (coronary artery bypass graft (CABG), mitral or aortic valve surgery)	≤ 5 mg/day 3137 (29.1%) 5–10 mg/day 1459 (13.5%) > 10 mg/day 461 (4.3%)	Charlson Comorbidity Score - Mean score of those on TNF = 2.7+/-2.8 *n* = 834 (23.7%) on TNF had diabetes. *n* = 717 (20.4%) on TNF had asthma/COPD	M = 72.5	NR	Adalimumab, Certolizumab, Etanercept, Golimumab, Infliximab	NR	Stop: *n* = 1724 Continue: *n* = 1311
Juo 2019 [23]	Diagnosis of Rheumatoid Arthritis (2 + ICD9 diagnosis codes of 714 at least 6 months apart) Any type of surgery	Chronic Steroid Use: Stop *n* = 3 (50%) Continue *n* = 92 (25%) Other use pattern *n* = 49 (27%)	Diabetes: Continue *n* = 96 (26%) Stop *n* = 3 (50%) Other use pattern *n* = 51 (29%) Charlson Comorbidity Index: Continue: 1.8 Stopped: 2.5 Other use pattern:1.9	Stop group M = 58.66 (SD = 7.61) Continue group M = 65.67 years (SD = 9.62)	Active smoking: Stop: *n* = 0 (0%) Continue: *n* = 100 (27%) Other use pattern: *n* = 46 (26%)	Combination of methotrexate and a TNF (Tumor Necrosis Factor) inhibitor: Adalimumab, Etanercept	NR	Stop: *n* = 6 Continue: *n* = 363 Other pattern use: *n* = 181
Ruyssen-Witrand 2005 & 2007 [10]	Outpatients and inpatients patients of a tertiary centre rheumatology unit who were treated with TNF alpha blockers Included orthopaedic, abdominal, gynaecological and other procedures	44% (56/127) of surgical procedures were performed on patients receiving concomitant corticosteroids	Diabetes Mellitus: Present in 3% (4/127) of the procedures.	M = 53	24% (31/127) were active smokers.	Etanercept Infliximab Adalimumab	NR	Stop: *n* = 36 Continue: *n* = 65
Ward 2020 [9]	RA diagnosis Coronary artery bypass surgery (CABG), vascular surgery, or open or laparoscopic bowel resection	Parenteral corticosteroids (per surgery): CABG: *n* = 16 (2.2%) Bowel resection: *n* = 24 (2.7%) Chronic prednisone use was noted in 14.6% to 20.4% of patients across the cohorts	Diabetes (per surgery) CABG: *n* = 337 (47.3%) Vascular Surgery: *n* = 108 (44.3%) Bowel resection: *n* = 240 (27.8%)	(CABG) M = 72.4 (SD = 7.2). Vascular Surgery M = 74.2 years (SD = 8.0) Bowel Resection M = 74.0 years (SD = 8.3).	Current smoker (Per surgery) CABG: *n* = 107 (15%) Vascular Surgery: *n* = 55 (22.5%) Bowel resection: *n* = 179 (20.8%)	Infliximab	NR	NR
Wendling 2005 [19]	- RA diagnosis Surgery types not specified	General steroid treatment in 41/50 patients receiving a mean of 8.2 mg/day of prednisone	NR	M = 54.6 years	NR	Infliximab (*n* = 26), Etanercept (*n* = 13), Adalimumab (*n* = 11)	NR	Stop: *n* = 18 (out of 50) Continue: *n* = 32 (out of 50)

BA - Biologic Agent, bDMARD - Biologic disease-modifying anti-rheumatic drugs, CI - Confidence Interval, COPD - Chronic Obstructive Pulmonary Disease, ICD-9 - International Classification of Diseases 9th Revision, M-mean, NR - None Reported, OR - Odds Ratio, PA/PsA - Psoriatic arthritis, RA - Rheumatoid Arthritis, SD - Standard deviation, SSTI - Skin and soft tissue infection, TNF - Tumour Necrosis Factor

### Outcomes

Due to the heterogeneity of the included studies, it wasn’t possible to combine the results in a meta-analysis and studies were therefore discussed narratively by outcome.

### Surgical site infection

Five studies [8, 10, 16, 19, 23] reported infections as an outcome, however, only three of these studies [8, 16, 23] compared infection rates between the stopping compared to the continuation of biologics groups. Of the five studies that reported on this outcome, four [8, 10, 16, 19] were judged to have serious risk of bias and one study [23] was judged to have a moderate risk of bias. Table 2 notes the definitions of the infections and the timeframe in which they were assessed.

**Table 2:** Definitions of infections used in included studies

Study	Outcome Definition	Timeframe assessed	SSI / SSTI / Composite
Juo 2019 [23]	Pneumonia, sepsis, UTI and wound infection	30 days post-operative	Composite
Elia 2020 [16]	Wound Infection	Whilst hospitalised	NR
Bakkour 2016 [8]	Wound Infection	NR	Composite
Wendling 2005 [19]	NR	NR	Composite
Ruyssen-Witrand 2007 [10]	Infection at the surgical site, either superficial or deep, within 1 month after surgery, or within 12 months for implant material (such as prosthesis)	30 days (12 months)	SSI

NR: Not Reported; SSI: Surgical Site Infection; Skin and Soft Tissue Infection; UTI: Urinary Tract Infection;

### Wound infection

Bakkour et al. (2016) reported that 4 people out of a total of 20 (25%) had a wound infection post-surgery when they stopped their biologic compared to 2 people out of 57 (3.63%) in the continue group [8]. Elia et al., (2020) reported that 2 people out of a total of 20 (10%) had a wound infection during hospitalisation post operatively in the stop group compared to 1 out of 19 people (5.26%) in the group who continued their biologics [16].

### Composite infections

Juo et al., (2019) reported that 22 people out of a total of 363 (6.1%) had an infection recorded within 30 days of surgery when they continued their biologic during the perioperative period compared to zero (0/6) in the stop group [23].

Wendling et al., (2005) reported no major complications, including infections, in either the stop or continue biologic group [19].

### Surgical site infection

Ruyssen-Witrand et al. (2005) [10] collected surgical site infection data but did not report their results in enough detail to compare between the stop and continue groups.

### Delayed wound healing

Four studies [8, 10, 16, 19] reported on the outcome of delayed wound healing. Of these, two studies [8, 16] compared delayed wound healing rates between the stop and continue biologics groups, both of which were judged as having a serious risk of bias. There was very little information on how delayed wound healing was defined across the included studies. The definitions the studies report are listed in Table 3.

**Table 3:** Definitions of delayed wound healing in included studies

Study	Delayed wound healing definition used	TImeframe Assessed
Bakkour 2016 [8]	Delayed wound healing	NR
Ruyssen-Witrand 2007 [10]	dehiscence or delayed healing	2 months post-operative
Elia 2020 [16]	Delayed wound healing, or wound dehiscence requiring surgery.	NR
Wendling 2005 [19]	delay of wound healing of 1–2 weeks	NR

NR: Not reported

Two studies [8, 16] reported higher rates of delayed wound healing for procedures where biologics were stopped before surgery. Bakkour et al., (2016) reported 3 out of 20 (15%) people in the stop group had delayed wound healing compared to 1 person out of 57 (1.8%) in the group where biologics were continued prior to surgery, although this data also included wound infections [8]. Elia et al., (2020) found delayed wound healing in 4 out of a total of 20 (20%) people in the stop group compared to 2 people out of 19 (11%) in the group who continued their biologics [16].

In addition, Elia et al., found that 5.1% (*n* = 1) of their sample had wound dehiscence requiring surgery in the stop group compared to zero experiencing this outcome in the continue biologic group [16].

### Disease flare(s)

Three studies measured disease flare, all of which were judged as having a serious risk of bias [8, 16, 19]. Studies used different outcomes to measure disease flare including hospital admission secondary to flare [16] and increase in joint count and global assessment of > 20% [19]. One study did not describe this [8]. Two studies included a mixture of different orthopaedic and non-orthopaedic procedures and did not provide separate results by procedure [8, 19]. The third examined patients who had undergone posterior arthrodesis of the craniovertebral junction [16].

All three studies reported a higher rate of disease flare following procedures for which biologics were stopped before surgery [8, 16, 19]. Bakkour et al., (2016) reported that 30% of their sample had a disease flare when they stopped taking their biologic in the perioperative period compared to no one in the continuation group [8]. The second study reported that 2 people out of 20 (10%) required a hospital admission for a rheumatoid arthritis flare up after stopping their biologic compared to no one (*n* = 19) in the continue group [16]. Wendling et al., (2005) reported that 5 people out of a total of 18 (28%) had a flare up of their IA when they stopped their biologic compared to 1 person out of a total of 32 (3%) who continued their biologic, which was a statistically significant difference [19].

### Quality of Life

None of the included studies measured quality of life.

### Risk of bias in included studies

**Fig. 2:**
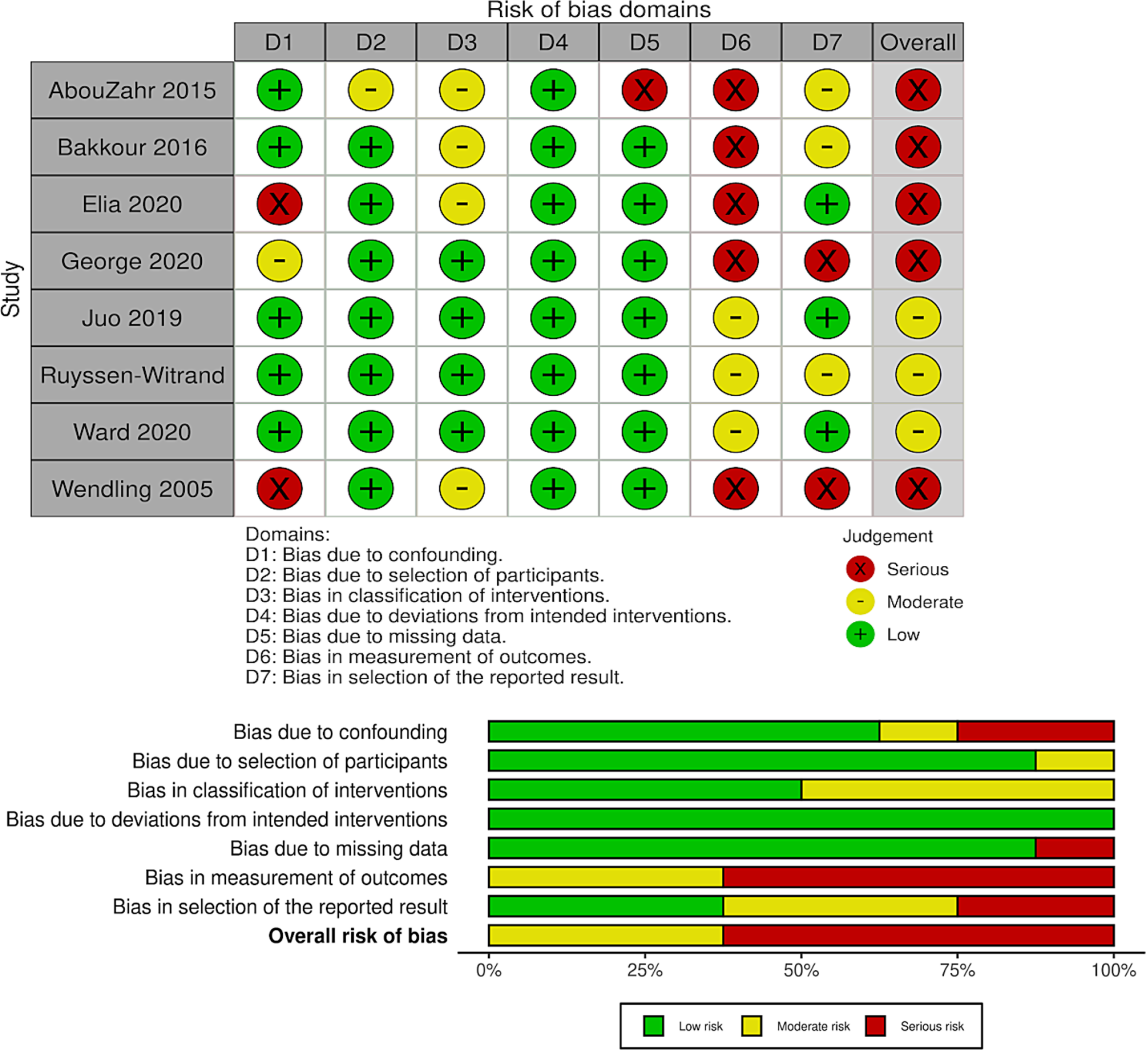
Risk of bias summary

The overall risk of bias was serious or moderate for all included studies, with 5 of the 8 studies being at serious risk of bias (Fig. 2). All studies were considered at low risk of bias in the ‘bias due to deviations from intended interventions’ domain, which is potentially due to the nature of the intervention. Due to the retrospective nature of these studies, the outcome of infections was measured by the treating clinician, resulting in the ‘bias in the measurement of outcomes’ domain being categorised as serious or moderate risk of bias for all studies.

## Discussion

This systematic review sought to evaluate whether continuing or stopping bDMARDs during the perioperative period affects SSIs, wound healing, or disease flares in patients with IA undergoing elective non-orthopaedic surgery. Overall, findings from the eight included studies, though limited in quality, provide some challenge to the prevailing assumption that continuation of biologic therapy increases perioperative infection risk and wound complications. Some studies indicated higher complication rates, specifically infections and flares, in patients who stopped biologic therapy compared to those who continued it, although the observational nature of the studies, the serious risk of bias and small comparator groups including small sample sizes overall limit the generalisability of the findings of this review.

Across studies assessing infections, there are conflicting findings with Bakkour et al., (2016) [8] reporting increased infection rates in patients who stopped their biologics (*n* = 4, 25%) compared to those who continued (*n* = 2, 3.63%%). Juo et al., (2019) meanwhile observed an increased rate of infection for those who continued their biologic (*n* = 22, 6.1%) compared to no infections in the stop group, though this finding must be interpreted cautiously due to the very small sample in the comparator group. These conflicting outcomes reflect both the methodological limitations of retrospective data, heterogeneity in the infection definitions used, and the likely role of confounding factors such as surgery type, disease severity, and comorbidities.

Similar patterns were seen in studies reporting delayed wound healing. Two studies showed higher rates of delayed healing or wound complications in patients who stopped their biologics, though the outcomes were inconsistently defined and often combined with infection-related data [8, 16]. The ambiguity in outcome definition and timing across studies underscores the need for standardised reporting and limits the interpretability of the results.

Disease flares were less commonly assessed, and when reported, were measured inconsistently without using validated outcome measures and with no standardisation in flare definitions. Therefore, due to the level of variability, it is not possible to directly compare disease flare outcomes across the included studies. The observational evidence does suggest increased flares in the stop group compared with the continue group, which aligns with prior evidence and current clinical guidelines indicating that interruption of biologic therapy increases the risk of disease flares, which can itself compromise postoperative recovery and quality of life [3, 4].

Several limitations of the included studies limit the strength of conclusions that can be made. No randomised controlled trials (RCTs) were identified and most included studies were retrospective cohorts which were evaluated as being at serious risk of bias. None of the included studies were adequately adjusted for confounding by indication, disease severity, or perioperative steroid use. Common issues also included selection bias and reliance on clinician-assessed outcomes without blinding. Heterogeneity in biologic agents and perioperative management protocols further complicates comparisons across studies. Included studies primarily included TNF-inhibitors with other biologics of alternate mechanisms of action being limited to only one or two included studies (e.g. abatacept, anakinra, ustekinumab). Some biologic groups such as IL-6 inhibitors, IL-17 inhibitors, and IL-23 p19 inhibitors were not included in any of these studies.

Despite following rigorous systematic review methods, this review faced several constraints. Most of the included studies enrolled mixed surgical populations, incorporating orthopaedic procedures without stratified outcome reporting. Excluding such studies would have resulted in substantial loss of available evidence, particularly given the absence of randomised trials and the limited volume of high-quality data in exclusively non-orthopaedic cohorts, therefore, it was not possible to isolate the impact of biologic continuation in strictly non-orthopaedic surgical contexts for IA patients. Finally, publication and language biases cannot be ruled out, though no language restrictions were applied to the searches. The review findings should therefore be interpreted cautiously in light of these limitations.

Current clinical guidelines recommend stopping biologics prior to surgery, based largely on consensus or evidence from orthopaedic populations [3, 4]. Several systematic reviews [24, 25] have identified that the type of surgery can affect the risks, types and timing of surgical site infections, thus evidence from orthopaedic-focussed studies may not be informative for non-orthopaedic surgery populations. This review highlights that such guidance may not be directly applicable to non-orthopaedic surgical settings. The absence of evidence of a clear, consistent association between biologic continuation and increased SSI or wound complications may call into question the routine withholding of these agents, especially given the demonstrated risks associated with disease flare.

There is an urgent need for high-quality RCTs to determine the safety and efficacy of biologic continuation in non-orthopaedic surgery populations. Ongoing studies such as PERISCOPE will help address this gap in orthopaedic contexts, but parallel research is needed across a broader range of surgical procedures. Additionally, future studies should use standardised definitions for perioperative outcomes and include quality of life assessments to support shared decision-making in clinical practice.

## Electronic Supplementary Material

Below is the link to the electronic supplementary material.

Supplementary Material 1

Supplementary Material 2

## Data Availability

All data generated or analysed during this study are included in this published article [and its supplementary information files].

## Abbreviations

bDMARDs: Biologic Disease-Modifying Anti-Rheumatic Drugs IA: Inflammatory Arthritis
IA: Inflammatory Arthiritis
PERISCOPE: PERI-operative biologic DMARD management: Stoppage or COntinuation during orthoPaEdic operations (the PERISCOPE trial)
PRISMA: Preferred Reporting Items for Systematic reviews and Meta-Analyses
PROSPERO: International Prospective Register of Systematic Reviews
RCTs: Randomised Controlled Trials
ROBINS-I: Risk Of Bias In Non-randomised Studies - of Interventions
SSIs: Surgical Site Infections
