# Supplementary material for: Perioperative biologic use and postoperative outcomes in patients with inflammatory arthritis: a systematic review
Source: BMC Rheumatol. 2026 Apr 30;10:50. doi: 10.1186/s41927-026-00650-y (PMC13274095; doi:10.1186/s41927-026-00650-y)
Supplement: Supplementary file 1 — Supplementary Material 1 [file 41927_2026_650_MOESM1_ESM.docx]

**Supplementary document 1: Search strategies**

**Ovid MEDLINE(R) ALL**

via Ovid <http://ovidsp.ovid.com/>

Date range searched: <1946 to April 22, 2024>

Date searched: 23 April 2024

Records retrieved: 1393

1 Arthritis, Rheumatoid/ (112387)

2 Caplan Syndrome/ (182)

3 Felty Syndrome/ (740)

4 Rheumatoid Nodule/ (977)

5 Rheumatoid Vasculitis/ (79)

6 ((arthriti* or polyarthriti*) adj2 r?eumat*).ti,ab,kw. (127567)

7 r?eumarthritis.ti,ab,kw. (5)

8 inflammatory arthritis.ti,ab,kw. (6552)

9 chronic articular r?eumatism.ti,ab,kw. (34)

10 ((caplan* or felty*) adj syndrom*).ti,ab,kw. (898)

11 (r?eumat* adj2 (nodul* or vasculit*)).ti,ab,kw. (2092)

12 Arthritis, Psoriatic/ (8557)

13 (psoria* adj2 (arthriti* or arthropath* or polyarthr*)).ti,ab,kw. (13991)

14 Spondylitis, Ankylosing/ (16902)

15 ((ankylo* or rheumat*) adj2 spondyl*).ti,ab,kw. (18611)

16 ((bechterew* or marie stru?mpell*) adj2 disease*).ti,ab,kw. (428)

17 Arthritis, Juvenile/ (11973)

18 ((arthriti* or oligoarthriti* or polyarthriti*) adj5 (child* or juvenile* or infant*)).ti,ab,kw. (14673)

19 ((juvenile onset or juvenile-onset) adj (still* adj disease*)).ti,ab,kw. (7)

20 or/1-19 (196695)

21 Biological Products/ (36652)

22 (biologic* adj4 (drug* or therap* or product*)).ti,ab,kw. (49417)

23 ((anti r?eumat* or antir?eumat* or anti-r?eumat*) adj3 (drug* or agent*)).ti,ab,kw. (11207)

24 bDMARD*.ti,ab,kw. (1496)

25 exp Antibodies, Monoclonal/ (283619)

26 (antibod* adj3 monoclonal).ti,ab,kw. (217150)

27 Tumor Necrosis Factor Inhibitors/ (2876)

28 Tumor Necrosis Factor-alpha/ai (16386)

29 Interleukin-6 Inhibitors/ (13)

30 Interleukin-6/ai (2059)

31 Interleukin-12/ai (592)

32 Interleukin-23/ai (327)

33 Interleukin-17/ai (974)

34 ((TNF* or tumo?r necrosis factor or IL-6 or interleukin-6 or IL-12 or interleukin-12 or IL-23 or interleukin-23 or IL-17 or interleukin-17) adj2 (inhibit* or block* or antagonist*)).ti,ab,kf,nm,rn. (37266)

35 Adalimumab/ (7194)

36 (adalimumab* or amjevita* or amgevita* or humira* or abrilada* or adaly* or amsparity* or cinnora* or cyltezo* or exemptia* or fyzoclad* or hadlima* or halimatoz* or hefiya* or hukyndra* or hulio* or hyrimoz* or idacio* or imraldi* or kromeya* or libmyris* or mabura* or qletli* or raheara* or solymbic* or sulinno* or trudexa* or yuflyma* or yusimry*).ti,ab,kf,nm,rn. (11615)

37 Etanercept/ (6584)

38 (etanercept* or enbrel* or embrel* or erelzi* or avent* or benepali* or enerceptan* or erelzi* or etacept* or etanar* or eticovo* or infinitam* or lifmior* or nepexto* or opinercept* or reumatocept* or tunex* or yisaipu*).ti,ab,kf,nm,rn. (10804)

39 (golimumab* or shinponi* or simponi*).ti,ab,kf,nm,rn. (1710)

40 Certolizumab Pegol/ (756)

41 (certolizumab* or cimzia* or simziya* or xcimzane*).ti,ab,kf,nm,rn. (1667)

42 Infliximab/ (12321)

43 (infliximab* or inflectra* or remicade* or renflexis* or avakine* or avsola* or flixabi* or remsima* or revellex* or zessly*).ti,ab,kf,nm,rn. (18310)

44 Abatacept/ (3437)

45 (abatacept* or belatacept* or nulojix* or orencia* or CTLA4-Ig).ti,ab,kf,nm,rn. (5094)

46 (tocilizumab* or actemra* or atlizumab* or lusinex* or roactemra*).ti,ab,kf,nm,rn. (7083)

47 (sarilumab* or kevzara*).ti,ab,kf,nm,rn. (381)

48 Ustekinumab/ (1926)

49 (ustekinumab* or stelara*).ti,ab,kf,nm,rn. (3478)

50 (secukinumab* or cosentyx* or scapho*).ti,ab,kf,nm,rn. (10021)

51 (ixekizumab* or taltz*).ti,ab,kf,nm,rn. (1127)

52 (guselkumab* or tremfya*).ti,ab,kf,nm,rn. (709)

53 (risankizumab* or skyrizi*).ti,ab,kf,nm,rn. (499)

54 Rituximab/ (19428)

55 (rituximab* or blitzima* or halpryza* or mabthera* or riabni* or ritemvia* or ritucad* or ritumax* or rituxan* or rituxin* or rituzena* or rixathon* or riximyo* or ruxience* or truxima* or tuxella*).ti,ab,kf,nm,rn. (32405)

56 or/21-55 (520278)

57 20 and 56 (33066)

58 Perioperative Care/ (16240)

59 Perioperative Medicine/ (158)

60 Perioperative Nursing/ (7000)

61 Perioperative Period/ (4057)

62 Intraoperative Care/ (17719)

63 Intraoperative Period/ (14653)

64 su.fs. (2310521)

65 (surger* or surgical* or operat* or procedur* or peri-op* or periop* or intra-op* or intraop* or peri-procedur* or periprocedur* or perisurg* or peri-surg*).ti,ab,kw. (4144447)

66 or/58-65 (5129129)

67 57 and 66 (1555)

68 exp animals/ not humans.sh. (5214079)

69 67 not 68 (1531)

70 limit 69 to yr="2000 -Current" (1393)

**Key:**

/ or .sh. = indexing term (Medical Subject Heading: MeSH)

exp = exploded indexing term (MeSH)

/ai = MeSH term with subheading for antagonists and inhibitors

* = truncation

? = wildcard for 0-1 letters

ti,ab,kw = terms in either title, abstract, or keyword fields

kf,nm,rn = keyword heading word, name of substance word, registry number / name of substance fields

su.fs = surgery as a floating subheading

adj3 = terms within three words of each other (any order)

**Embase**

via Ovid <http://ovidsp.ovid.com/>

Date range searched: <1974 to 2024 April 22>

Date searched: 23 April 2024

Records retrieved: 3323

1 rheumatoid arthritis/ (220344)

2 Felty syndrome/ (918)

3 rheumatoid nodule/ (1752)

4 rheumatoid vasculitis/ (391)

5 ((arthriti* or polyarthriti*) adj2 r?eumat*).ti,ab,kw. (190682)

6 r?eumarthritis.ti,ab,kw. (8)

7 inflammatory arthritis.ti,ab,kw. (12869)

8 chronic articular r?eumatism.ti,ab,kw. (9)

9 ((caplan* or felty*) adj syndrom*).ti,ab,kw. (877)

10 (r?eumat* adj2 (nodul* or vasculit*)).ti,ab,kw. (3131)

11 psoriatic arthritis/ (32394)

12 (psoria* adj2 (arthriti* or arthropath* or polyarthr*)).ti,ab,kw. (28042)

13 ankylosing spondylitis/ (32774)

14 ((ankylo* or rheumat*) adj2 spondyl*).ti,ab,kw. (30054)

15 ((bechterew* or marie stru?mpell*) adj2 disease*).ti,ab,kw. (362)

16 juvenile rheumatoid arthritis/ (24800)

17 ((arthriti* or oligoarthriti* or polyarthriti*) adj5 (child* or juvenile* or infant*)).ti,ab,kw. (24115)

18 ((juvenile onset or juvenile-onset) adj (still* adj disease*)).ti,ab,kw. (9)

19 or/1-18 (321566)

20 *biological product/ (16357)

21 (biologic* adj4 (drug* or therap* or product*)).ti,ab,kw. (77785)

22 ((anti r?eumat* or antir?eumat* or anti-r?eumat*) adj3 (drug* or agent*)).ti,ab,kw. (18907)

23 bDMARD*.ti,ab,kw. (4828)

24 *monoclonal antibody/ (80742)

25 (antibod* adj3 monoclonal).ti,ab,kw. (278672)

26 *tumor necrosis factor inhibitor/ (5397)

27 ((TNF* or tumo?r necrosis factor or IL-6 or interleukin-6 or IL-12 or interleukin-12 or IL-23 or interleukin-23 or IL-17 or interleukin-17) adj2 (inhibit* or block* or antagonist*)).ti,ab,kf,du,dy,tn. (73210)

28 adalimumab/ (47976)

29 (adalimumab* or amjevita* or amgevita* or humira* or abrilada* or adaly* or amsparity* or cinnora* or cyltezo* or exemptia* or fyzoclad* or hadlima* or halimatoz* or hefiya* or hukyndra* or hulio* or hyrimoz* or idacio* or imraldi* or kromeya* or libmyris* or mabura* or qletli* or raheara* or solymbic* or sulinno* or trudexa* or yuflyma* or yusimry*).ti,ab,kf,du,dy,tn. (49064)

30 etanercept/ (38934)

31 (etanercept* or enbrel* or embrel* or erelzi* or avent* or benepali* or enerceptan* or erelzi* or etacept* or etanar* or eticovo* or infinitam* or lifmior* or nepexto* or opinercept* or reumatocept* or tunex* or yisaipu*).ti,ab,kf,du,dy,tn. (53465)

32 golimumab/ (10702)

33 (golimumab* or shinponi* or simponi*).ti,ab,kf,du,dy,tn. (10906)

34 certolizumab pegol/ (9749)

35 (certolizumab* or cimzia* or simziya* or xcimzane*).ti,ab,kf,du,dy,tn. (10456)

36 infliximab/ (65458)

37 (infliximab* or inflectra* or remicade* or renflexis* or avakine* or avsola* or flixabi* or remsima* or revellex* or zessly*).ti,ab,kf,du,dy,tn. (66959)

38 abatacept/ (13236)

39 (abatacept* or belatacept* or nulojix* or orencia* or CTLA4-Ig).ti,ab,kf,du,dy,tn. (16639)

40 tocilizumab/ (29107)

41 (tocilizumab* or actemra* or atlizumab* or lusinex* or roactemra*).ti,ab,kf,du,dy,tn. (30394)

42 sarilumab/ (2255)

43 (sarilumab* or kevzara*).ti,ab,kf,du,dy,tn. (2307)

44 ustekinumab/ (13919)

45 (ustekinumab* or stelara*).ti,ab,kf,du,dy,tn. (14225)

46 secukinumab/ (7854)

47 (secukinumab* or cosentyx* or scapho*).ti,ab,kf,du,dy,tn. (16670)

48 ixekizumab/ (3988)

49 (ixekizumab* or taltz*).ti,ab,kf,du,dy,tn. (4085)

50 guselkumab/ (2627)

51 (guselkumab* or tremfya*).ti,ab,kf,du,dy,tn. (2686)

52 risankizumab/ (1834)

53 (risankizumab* or skyrizi*).ti,ab,kf,du,dy,tn. (1878)

54 rituximab/ (114837)

55 (rituximab* or blitzima* or halpryza* or mabthera* or riabni* or ritemvia* or ritucad* or ritumax* or rituxan* or rituxin* or rituzena* or rixathon* or riximyo* or ruxience* or truxima* or tuxella*).ti,ab,kf,du,dy,tn. (122471)

56 or/20-55 (659827)

57 19 and 56 (79923)

58 perioperative care/ (2868)

59 perioperative medicine/ (811)

60 perioperative nursing/ (6268)

61 perioperative period/ (67147)

62 intraoperative period/ (53808)

63 su.fs. (2433703)

64 (surger* or surgical* or operat* or procedur* or peri-op* or periop* or intra-op* or intraop* or peri-procedur* or periprocedur* or perisurg* or peri-surg*).ti,ab,kw. (5462593)

65 or/58-64 (6538261)

66 57 and 65 (5524)

67 (rat or rats or mouse or mice or swine or porcine or murine or sheep or lambs or pigs or piglets or rabbit or rabbits or cat or cats or dog or dogs or cattle or bovine or monkey or monkeys or trout or marmoset$).ti,ot. and animal experiment/ (1249030)

68 Animal experiment/ not (human experiment/ or human/) (2626344)

69 67 or 68 (2699337)

70 66 not 69 (5483)

71 (comment or conference or letter or editorial or note).pt. (9002753)

72 (letter or comment*).ti. (244214)

73 letter/ (1236850)

74 or/71-73 (9069717)

75 70 not 74 (3471)

76 limit 75 to yr="2000 -Current" (3323)

**Key:**

/ or .sh. = indexing term (Emtree Subject Heading)

exp = exploded indexing term (Emtree)

* before an Emtree heading = focussed Emtree heading

* = truncation

ti,ab,kw = terms in either title, abstract or keyword fields

kf,du,dy,tn. = keyword heading word, drug index terms, drug index terms word, drug trade name fields

adj3 = terms within three words of each other (any order)

? = wildcard for 0-1 letters

su.fs = surgery as a floating subheading

pt = publication type

ot = original title

**Cochrane Central Register of Controlled Trials (CENTRAL)**

via Wiley <http://onlinelibrary.wiley.com/>

Date range searched: Issue 3 of 12, March 2024

Date searched: 23 April 2024

Records retrieved: 2142

#1 [mh ^"Arthritis, Rheumatoid"] 7606

#2 [mh ^"Caplan Syndrome"] 0

#3 [mh ^"Felty Syndrome"] 0

#4 [mh ^"Rheumatoid Nodule"] 11

#5 [mh ^"Rheumatoid Vasculitis"] 0

#6 ((arthriti* or polyarthriti*) NEAR/2 r?eumat*):ti,ab,kw 19205

#7 r?eumarthritis:ti,ab,kw 0

#8 "inflammatory arthritis":ti,ab,kw 520

#9 ("chronic articular" NEXT r?eumatism):ti,ab,kw 13

#10 ((caplan* or felty*) NEAR/1 syndrom*):ti,ab,kw 6

#11 (r?eumat* NEAR/2 (nodul* or vasculit*)):ti,ab,kw 75

#12 [mh ^"Arthritis, Psoriatic"] 732

#13 (psoria* NEAR/2 (arthriti* or arthropath* or polyarthr*)):ti,ab,kw 3157

#14 [mh ^"Spondylitis, Ankylosing"] 919

#15 ((ankylo* or rheumat*) NEAR/2 spondyl*):ti,ab,kw 2823

#16 ((bechterew* or marie NEXT stru?mpell*) NEAR/2 disease*):ti,ab,kw 7

#17 [mh ^"Arthritis, Juvenile"] 449

#18 ((arthriti* or oligoarthriti* or polyarthriti*) NEAR/5 (child* or juvenile* or infant*)):ti,ab,kw 1159

#19 ("juvenile onset" NEAR/1 (still* NEAR/1 disease*)):ti,ab,kw 0

#20 {OR #1-#19} 24385

#21 [mh ^"Biological Products"] 729

#22 (biologic* NEAR/4 (drug* or therap* or product*)):ti,kw 2306

#23 ((anti NEXT r?eumat* or antir?eumat*) NEAR/3 (drug* or agent*)):ti,kw 3797

#24 bDMARD*:ti,ab,kw 649

#25 [mh ^"Antibodies, Monoclonal"[mj]] 81

#26 (antibod* NEAR/3 monoclonal):ti,kw 14635

#27 [mh ^"Tumor Necrosis Factor Inhibitors"] 164

#28 [mh ^"Tumor Necrosis Factor-alpha"/ai] 817

#29 [mh ^"Interleukin-6 Inhibitors"] 0

#30 [mh ^"Interleukin-6"/ai] 71

#31 [mh ^"Interleukin-12"/ai] 21

#32 [mh ^"Interleukin-23"/ai] 37

#33 [mh ^"Interleukin-17"/ai] 85

#34 ((TNF* or "tumor necrosis factor" or "tumour necrosis factor" or "tumor necrosis factors" or "tumour necrosis factors" or "IL-6" or "interleukin-6" or "IL-12" or "interleukin-12" or "IL-23" or "interleukin-23" or "IL-17" or "interleukin-17") NEAR/2 (inhibit* or block* or antagonist*)):ti,ab,kw 3564

#35 [mh ^Adalimumab] 1159

#36 (adalimumab* or amjevita* or amgevita* or humira* or abrilada* or adaly* or amsparity* or cinnora* or cyltezo* or exemptia* or fyzoclad* or hadlima* or halimatoz* or hefiya* or hukyndra* or hulio* or hyrimoz* or idacio* or imraldi* or kromeya* or libmyris* or mabura* or qletli* or raheara* or solymbic* or sulinno* or trudexa* or yuflyma* or yusimry*):ti,ab,kw 4009

#37 [mh ^Etanercept] 1018

#38 (etanercept* or enbrel* or embrel* or erelzi* or avent* or benepali* or enerceptan* or erelzi* or etacept* or etanar* or eticovo* or infinitam* or lifmior* or nepexto* or opinercept* or reumatocept* or tunex* or yisaipu*):ti,ab,kw 3160

#39 (golimumab* or shinponi* or simponi*):ti,ab,kw 800

#40 [mh ^"Certolizumab Pegol"] 221

#41 (certolizumab* or cimzia* or simziya* or xcimzane*):ti,ab,kw 781

#42 [mh ^Infliximab] 1067

#43 (infliximab* or inflectra* or remicade* or renflexis* or avakine* or avsola* or flixabi* or remsima* or revellex* or zessly*):ti,ab,kw 2717

#44 [mh ^Abatacept] 418

#45 (abatacept* or belatacept* or nulojix* or orencia* or "CTLA4-Ig"):ti,ab,kw 1229

#46 (tocilizumab* or actemra* or atlizumab* or lusinex* or roactemra*):ti,ab,kw 1694

#47 (sarilumab* or kevzara*):ti,ab,kw 332

#48 [mh ^Ustekinumab] 341

#49 (ustekinumab* or stelara*):ti,ab,kw 1193

#50 (secukinumab* or cosentyx* or scapho*):ti,ab,kw 1412

#51 (ixekizumab* or taltz*):ti,ab,kw 660

#52 (guselkumab* or tremfya*):ti,ab,kw 621

#53 (risankizumab* or skyrizi*):ti,ab,kw 298

#54 [mh ^Rituximab] 1985

#55 (rituximab* or blitzima* or halpryza* or mabthera* or riabni* or ritemvia* or ritucad* or ritumax* or rituxan* or rituxin* or rituzena* or rixathon* or riximyo* or ruxience* or truxima* or tuxella*):ti,ab,kw 6120

#56 {OR #21-#55} 37337

#57 #20 and #56 10205

#58 [mh ^"Perioperative Care"] 1333

#59 [mh ^"Perioperative Medicine"] 1

#60 [mh ^"Perioperative Nursing"] 79

#61 [mh ^"Perioperative Period"] 365

#62 [mh ^"Intraoperative Care"] 1905

#63 [mh ^"Intraoperative Period"] 1466

#64 (surger* or surgical* or operat* or procedur*):ti,ab,kw 586707

#65 (periop* or intraop* or periprocedur* or perisurg*):ti,ab,kw 62435

#66 {OR #58-#65} 592146

#67 #57 and #66 2185

#68 #57 and #66 with Publication Year from 2000 to 2024, in Trials 2142

**Key:**

mh = explode indexing term (MeSH)

mh ^ = unexploded indexing term (MeSH)

[mj] = focussed MeSH heading

/ai = MeSH term with subheading for antagonists and inhibitors

* = truncation or multiple additional characters within a word

ti,ab,kw = terms in either title or abstract or keyword fields

near/3 = terms within three words of each other (any order)

next = terms are next to each other.

? = wildcard for 0-1 additional characters

**CINAHL Complete**

via EBSCO (https://web.s.ebscohost.com/)

Date range: Inception - Current

Date searched: 23 April 2024

Records retrieved: 358

S57 S19 AND S48 AND S55 Limiters - Publication Date: 20000101-20240431 (358)

S56 S19 AND S48 AND S55 (365)

S55 S49 OR S50 OR S51 OR S52 OR S53 OR S54 (792,625)

S54 TI (surger* or surgical* or operat* or procedur* or peri-op* or periop* or intra-op* or intraop* or peri-procedur* or periprocedur* or perisurg* or peri-surg*) OR AB (surger* or surgical* or operat* or procedur* or peri-op* or periop* or intra-op* or intraop* or peri-procedur* or periprocedur* or perisurg* or peri-surg*) (778,864)

S53 (MH "Intraoperative Period") (4,786)

S52 (MH "Intraoperative Care") (5,922)

S51 (MH "Perioperative Nursing") (17,019)

S50 (MH "Perioperative Medicine") (36)

S49 (MH "Perioperative Care") (12,573)

S48 S20 OR S21 OR S22 OR S23 OR S24 OR S25 OR S26 OR S27 OR S28 OR S29 OR S30 OR S31 OR S32 OR S33 OR S34 OR S35 OR S36 OR S37 OR S38 OR S39 OR S40 OR S41 OR S42 OR S43 OR S44 OR S45 OR S46 OR S47 (74,532)

S47 TI (rituximab* or blitzima* or halpryza* or mabthera* or riabni* or ritemvia* or ritucad* or ritumax* or rituxan* or rituxin* or rituzena* or rixathon* or riximyo* or ruxience* or truxima* or tuxella*) OR AB (rituximab* or blitzima* or halpryza* or mabthera* or riabni* or ritemvia* or ritucad* or ritumax* or rituxan* or rituxin* or rituzena* or rixathon* or riximyo* or ruxience* or truxima* or tuxella*) (6,998)

S46 TI (risankizumab* or skyrizi*) OR AB (risankizumab* or skyrizi*) (140)

S45 TI (guselkumab* or tremfya*) OR AB (guselkumab* or tremfya*) (239)

S44 TI (ixekizumab* or taltz*) OR AB (ixekizumab* or taltz*) (325)

S43 TI (secukinumab* or cosentyx* or scapho*) OR AB (secukinumab* or cosentyx* or scapho*) (1,797)

S42 TI (ustekinumab* or stelara*) OR AB (ustekinumab* or stelara*) (799)

S41 TI (sarilumab* or kevzara*) OR AB (sarilumab* or kevzara*) (108)

S40 TI (tocilizumab* or actemra* or atlizumab* or lusinex* or roactemra*) OR AB (tocilizumab* or actemra* or atlizumab* or lusinex* or roactemra*) (1,816)

S39 TI (abatacept* or belatacept* or nulojix* or orencia* or CTLA4-Ig) OR AB (abatacept* or belatacept* or nulojix* or orencia* or CTLA4-Ig) (825)

S38 TI (infliximab* or inflectra* or remicade* or renflexis* or avakine* or avsola* or flixabi* or remsima* or revellex* or zessly*) OR AB (infliximab* or inflectra* or remicade* or renflexis* or avakine* or avsola* or flixabi* or remsima* or revellex* or zessly*) (3,507)

S37 TI (certolizumab* or cimzia* or simziya* or xcimzane*) OR AB (certolizumab* or cimzia* or simziya* or xcimzane*) (437)

S36 TI (golimumab* or shinponi* or simponi*) OR AB (golimumab* or shinponi* or simponi*) (503)

S35 TI (etanercept* or enbrel* or embrel* or erelzi* or avent* or benepali* or enerceptan* or erelzi* or etacept* or etanar* or eticovo* or infinitam* or lifmior* or nepexto* or opinercept* or reumatocept* or tunex* or yisaipu*) OR AB (etanercept* or enbrel* or embrel* or erelzi* or avent* or benepali* or enerceptan* or erelzi* or etacept* or etanar* or eticovo* or infinitam* or lifmior* or nepexto* or opinercept* or reumatocept* or tunex* or yisaipu*) (2,610)

S34 TI (adalimumab* or amjevita* or amgevita* or humira* or abrilada* or adaly* or amsparity* or cinnora* or cyltezo* or exemptia* or fyzoclad* or hadlima* or halimatoz* or hefiya* or hukyndra* or hulio* or hyrimoz* or idacio* or imraldi* or kromeya* or libmyris* or mabura* or qletli* or raheara* or solymbic* or sulinno* or trudexa* or yuflyma* or yusimry*) OR AB (adalimumab* or amjevita* or amgevita* or humira* or abrilada* or adaly* or amsparity* or cinnora* or cyltezo* or exemptia* or fyzoclad* or hadlima* or halimatoz* or hefiya* or hukyndra* or hulio* or hyrimoz* or idacio* or imraldi* or kromeya* or libmyris* or mabura* or qletli* or raheara* or solymbic* or sulinno* or trudexa* or yuflyma* or yusimry*) (2,521)

S33 ((TNF* or tumo?r necrosis factor or IL-6 or interleukin-6 or IL-12 or interleukin-12 or IL-23 or interleukin-23 or IL-17 or interleukin-17) N2 (inhibit* or block* or antagonist*)) OR AB (TNF* or tumo?r necrosis factor or IL-6 or interleukin-6 or IL-12 or interleukin-12 or IL-23 or interleukin-23 or IL-17 or interleukin-17) N2 (inhibit* or block* or antagonist*)) (5,073)

S32 TI (antibod* N3 monoclonal) OR AB (antibod* N3 monoclonal) (12,229)

S31 TI bDMARD* OR AB bDMARD* (395)

S30 TI ((anti r?eumat* or antir?eumat* or anti-r?eumat*) N3 (drug* or agent*)) OR AB ((anti r?eumat* or antir?eumat* or anti-r?eumat*) N3 (drug* or agent*)) (2,831)

S29 (biologic* N4 (drug* or therap* or product*)) OR AB (biologic* N4 (drug* or therap* or product*)) (19,549)

S28 (MH "Rituximab") (2,559)

S27 (MH "Abatacept") (202)

S26 (MH "Infliximab") (1,762)

S25 (MH "Certolizumab Pegol") (53)

S24 (MH "Etanercept") (1,021)

S23 (MH "Adalimumab") (697)

S22 (MH "Tumor Necrosis Factor Inhibitors") (492)

S21 (MH "Antibodies, Monoclonal") (33,167)

S20 (MH "Biological Products") (7,987)

S19 S1 OR S2 OR S3 OR S4 OR S5 OR S6 OR S7 OR S8 OR S9 OR S10 OR S11 OR S12 OR S13 OR S14 OR S15 OR S16 OR S17 OR S18 (47,662)

S18 TI ((juvenile onset or juvenile-onset) N1 (still* N1 disease*)) OR AB ((juvenile onset or juvenile-onset) N1 (still* N1 disease*)) (25)

S17 TI ((arthriti* or oligoarthriti* or polyarthriti*) N5 (child* or juvenile* or infant*)) OR AB ((arthriti* or oligoarthriti* or polyarthriti*) N5 (child* or juvenile* or infant*)) (4,097)

S16 TI ((bechterew* or marie stru?mpell*) N2 disease*) OR AB ((bechterew* or marie stru?mpell*) N2 disease*) (3)

S15 TI ((ankylo* or rheumat*) N2 spondyl*) OR AB ((ankylo* or rheumat*) N2 spondyl*) (4,475)

S14 TI (psoria* N2 (arthriti* or arthropath* or polyarthr*)) OR AB (psoria* N2 (arthriti* or arthropath* or polyarthr*)) (4,200)

S13 TI (r?eumat* N2 (nodul* or vasculit*)) OR AB (r?eumat* N2 (nodul* or vasculit*)) (358)

S12 TI ((caplan* or felty*) N1 syndrom*) OR AB ((caplan* or felty*) N1 syndrom*) (69)

S11 chronic articular r?eumatism OR AB chronic articular r?eumatism (0)

S10 inflammatory arthritis OR AB inflammatory arthritis (3,893)

S9 TI r?eumarthritis OR AB r?eumarthritis (2)

S8 TI ((arthriti* or polyarthriti*) N2 r?eumat*) OR AB ((arthriti* or polyarthriti*) N2 r?eumat*) (27,775)

S7 (MH "Arthritis, Juvenile Rheumatoid") (3,600)

S6 (MH "Spondylitis, Ankylosing") (3,918)

S5 (MH "Arthritis, Psoriatic") (3,182)

S4 (MH "Rheumatoid Nodule") (54)

S3 (MH "Felty's Syndrome") (32)

S2 (MH "Caplan Syndrome") (1)

S1 (MH "Arthritis, Rheumatoid") (25,614)

**Key:**

MH = indexing term (Medical Subject Heading: MeSH)

*  = truncation

TI OR AB = terms in either title or abstract fields

N3 = terms within three words of each other

? = wildcard for 0-1 letters

**International HTA database**

via <https://database.inahta.org/>

Date range searched: Inception - 23 April 2024

Date searched: 23 April 2024

Records retrieved: 268

All Fields: (arthriti* OR rheumat* OR reumat* OR arthropath* OR polyarthr* OR ankylosing spondylitis OR oligoarthriti*) AND (biologic OR drug* OR product* OR agent* OR bDMARD* OR TNF* OR adalimumab OR etanercept OR golimumab OR certolizumab OR infliximab OR abatacept OR tocilizumab OR sarilumab OR ustekinumab OR secukinumab OR ixekizumab OR guselkumab OR risankizumab OR rituximab OR TNF* OR tumor necrosis factor OR tumour necrosis factor OR IL-6 OR interleukin-6 OR IL-12 OR interleukin-12 OR IL-23 OR interleukin-23 OR IL-17 OR interleukin-17)

Publication Year 2000 – 2024

= 268

**Key:**

* = truncation

**ClinicalTrials.gov**

via <https://clinicaltrials.gov/>

Date searched: 23 April 2024

Records retrieved: 1116

Advanced search screen used. 6 separate searches were used, retrieving 1116 records in total, which were imported into EndNote 21 and deduplicated.

Condition/Disease: Arthritis

Other Terms: (drug OR product OR agent OR bDMARD OR TNF) AND (surgery OR perisurgical OR periprocedural OR perioperative OR intraoperative) = 844

Condition OR Disease: (Arthritis)

Other Terms: (drug OR product OR agent OR bDMARD OR TNF) AND (peri-surgical OR peri-procedural OR peri-operative OR intra-operative) = 219

Condition OR Disease: (Arthritis)

Other Terms: (adalimumab OR etanercept OR golimumab OR certolizumab OR infliximab OR abatacept OR tocilizumab) AND (peri-surgical OR peri-procedural OR peri-operative OR intra-operative) = 5

Condition OR Disease: (Arthritis)

Other Terms: (sarilumab OR ustekinumab OR secukinumab OR ixekizumab OR guselkumab OR risankizumab) AND (peri-surgical OR peri-procedural OR peri-operative OR intra-operative) = 0

Condition OR Disease: (Arthritis)

Other Terms: (adalimumab OR etanercept OR golimumab OR certolizumab OR infliximab OR abatacept OR tocilizumab) AND (surgery OR perisurgical OR periprocedural OR perioperative OR intraoperative) = 44

Condition OR Disease: (Arthritis)

Other Terms: (sarilumab OR ustekinumab OR secukinumab OR ixekizumab OR guselkumab OR risankizumab) AND (surgery OR perisurgical OR periprocedural OR perioperative OR intraoperative) = 4

**WHO ICTRP**

via <https://trialsearch.who.int/>

Date searched: 23 April 2024

Records retrieved: 29

Advanced search screen used. 6 separate searches were used, retrieving 29 records in total, which were imported into EndNote 21 and deduplicated.

Title: (surgery OR perisurgical OR periprocedural OR perioperative OR intraoperative)

Condition: (Arthritis)

Intervention: (drug OR product OR agent OR bDMARD OR TNF)

Recruitment status: ALL

26 trials found

Title: (peri-surgical OR peri-procedural OR peri-operative OR intra-operative)

Condition: (Arthritis)

Intervention: (drug OR product OR agent OR bDMARD OR TNF)

Recruitment status: ALL

3 trials found

Title: (peri-surgical OR peri-procedural OR peri-operative OR intra-operative)

Condition: (Arthritis)

Intervention: (adalimumab OR etanercept OR golimumab OR certolizumab OR infliximab OR abatacept OR tocilizumab)

Recruitment status: ALL

0 trials found

Title: (peri-surgical OR peri-procedural OR peri-operative OR intra-operative)

Condition: (Arthritis)

Intervention: (sarilumab OR ustekinumab OR secukinumab OR ixekizumab OR guselkumab OR risankizumab)

Recruitment status: ALL

0 trials found

Title: (surgery OR perisurgical OR periprocedural OR perioperative OR intraoperative)

Condition: (Arthritis)

Intervention: (adalimumab OR etanercept OR golimumab OR certolizumab OR infliximab OR abatacept OR tocilizumab)

Recruitment status: ALL

0 trials found

Title: (surgery OR perisurgical OR periprocedural OR perioperative OR intraoperative)

Condition: (Arthritis)

Intervention: (sarilumab OR ustekinumab OR secukinumab OR ixekizumab OR guselkumab OR risankizumab)

Recruitment status: ALL

0 trials found
