# Supplementary material for: Perioperative biologic use and postoperative outcomes in patients with inflammatory arthritis: a systematic review
Source: BMC Rheumatol. 2026 Apr 30;10:50. doi: 10.1186/s41927-026-00650-y (PMC13274095; doi:10.1186/s41927-026-00650-y)
Supplement: Supplementary file 2 — Supplementary Material 2 [file 41927_2026_650_MOESM2_ESM.docx]

**Appendix1: Excluded Studies at Full-text Screening**

| **Author - Year** | **Title** | **Reason for Exclusion** |
| --- | --- | --- |
| Gaudiani 2021 | The association of preoperative TNF-alpha inhibitor use and reoperation rates in spinal fusion surgery | Wrong intervention |
| Mori 2008 | Delayed spinal infection after laminectomy in a patient with rheumatoid arthritis interruptedly exposed to anti-tumor necrosis factor alpha agents | Wrong comparator |
| Gluck 2007 | Do DMARDs contibute to impaired healing after surgery? Should they be discontinued before surgery?. [German]Tragen basistherapeutika in der therapie rheumatischer erkrankungen zu wundheilungsstorungen bei? Sollten sie perioperativ abgesetzt werden? | Wrong study design |
| Gagne 2009 | Effect of immunosuppression on patients undergoing bariatric surgery | Wrong intervention |
| Ravi 2019 | Elucidating the Risks and Benefits of Withholding Biologics to Optimize Surgical Outcomes | Orthopaedic surgery |
| Khanna 2015 | The impact of steroids, methotrexate, and biologics on clinical and radiographic outcomes in patients with rheumatoid arthritis undergoing fusions at the craniovertebral junction | Wrong intervention |
| Tsai 2015 | Implications of Rheumatic Disease and Biological Response-Modifying Agents in Plastic Surgery | Wrong study design |
| Makino 2014 | Late-onset deep surgical-site infection after posterior lumbar interbody fusion in a patient treated with tocilizumab unusual changes in inflammatory markers | Wrong intervention |
| Rehart 2007 | Modern disease modifying drugs in rheumatoid arthritis in the perioperative period: Patients with rheumatic diseases under therapy with methotrexate, Leflunomide or TNF-alpha blockers in the perioperative period. [German]Moderne rheumatische basistherapie | Wrong patient population |
|  | An Observational Study to Describe in Routine Clinical Practice the Treatment Patterns of Usage of Biological DMARDs in RA Patients | Wrong comparator |
| Mamaril-Davis 2022 | Perioperative management of disease-modifying antirheumatic drugs for patients undergoing elective spine surgery: a systematic review | Wrong study design |
| George 2019 | Perioperative management of immunosuppression in patients with rheumatoid arthritis | Wrong study design |
| Zakaryan 2020 | Perioperative Management of Patients With Ankylosing Spondylitis Undergoing Spine Surgery | Wrong study design |
| Goupille 2007 | Perioperative management of patients with rheumatoid arthritis treated with TNF-alpha blocking agents...Semin Arthritis Rheum. 2007 Apr36(5):278-86 | Orthopaedic surgery |
| Thorsness 2012 | Perioperative management of rheumatoid medications | Wrong study design |
| Kostuj 2017 | Pilot study for the registry of complications in rheumatic diseases from the German Society of Surgery (DGORh): Evaluation of methods and data from the first 1000 patients | Orthopaedic surgery |
| Koyama 2016 | Postoperative Surgical Infection After Spinal Surgery in Rheumatoid Arthritis | Wrong intervention |
| Godot 2013 | Safety of surgery after rituximab therapy in 133 patients with rheumatoid arthritis: data from the autoimmunity and rituximab registry | Wrong intervention |
| Latourte 2017 | Safety of surgery in patients with rheumatoid arthritis treated by abatacept: data from the French Orencia in Rheumatoid Arthritis Registry | Wrong comparator |
| Morel 2020 | Safety of surgery in patients with rheumatoid arthritis treated with tocilizumab: data from the French (REGistry -RoAcTEmra) Regate registry | Wrong comparator |
| Wendling 2007 | Surgery in rheumatoid arthritis patients under anti-TNF-alpha therapy: what is the risk? | Wrong study design |
| Nishizaki 2024 | Targeted Therapies, Biologics, and Immunotherapy in the Neoadjuvant and Adjuvant Settings: Perioperative Risks | Wrong study design |
| Joo 2022 | Use of Disease-modifying Antirheumatic Drugs After Cancer Diagnosis in Rheumatoid Arthritis Patients | Wrong outcomes |
| Fields 2021 | Cervical Spinal Fusion in Adult Patients With Rheumatoid Arthritis <i>A National Analysis of Complications and 90</i>-<i>day Readmissions</i> | Wrong intervention |
| Atzeni 2018 | Conventional and Biological DMARDs in Systemic Rheumatic Diseases: Perioperative Risk/Benefit Management | Wrong study design |
| Wendling 2007 | Do patients with RA receiving anti-TNF agents have an increased risk of surgical site infections? | Orthopaedic surgery |
| Cohen 2023 | How to Manage Targeted Immune Suppressants (Biologics and Oral Small-molecule Drugs) Perioperatively for Inflammatory Bowel Disease and non-Inflammatory Bowel Disease surgery | Wrong study design |
| DenBroeder 2005 | Infectious complications in elective surgery in RA patients in the anti-TNF ERA: A retrospective study | Wrong patient population |
| Shergy 2005 | Infliximab and its impact on surgical outcomes in patients with rheumatoid arthritis | Full Text not retreivable |
| Kunimi 2021 | Intraocular surgery under adalimumab therapy in patients with refractory uveitis: a single center study of 23 eyes | Wrong patient population |
| Crawford 2008 | Lumbar fusion outcomes in patients with rheumatoid arthritis | Wrong intervention |
| Takakubo 2010 | Peri- and postoperative status of rheumatoid arthritis patients receiving biologic disease-modifying antirheumatic drugs | Unable to translate |
| Tsuneyoshi 2009 | Perioperative Management of Rheumatoid Arthritis Patients Treated with Biological Agents | Unable to translate |
| Kaufmann 2010 | Pre- and Postoperative Management of Patients Receiving Rituximab Therapy | Orthopaedic surgery |
| Ko 2009 | TNF Inhibitor Use during the Perioperative Period | Unable to translate |
| Rivera 2012 | Ustekinumab en situaciones especiales: embarazo, interrupciones temporales (vacunaciones, cirugÃ­a) y otros | Wrong study design |
| Galiano Mejias et al., 2016 | Management of biologic therapt in moderate to severe Psoriasis in surgical patients: Data from the Spanish Biobadaderm Registry | Wrong patient population |
| Nguyen et al., 2021 | Assessing the incidence of skin and soft tissue infection in patients on biologics | Patient population not defined |
